# Diet for the Fat

**Published:** 1899-04-15

**Authors:** 


### DIET FOR THE FAT.

Sometimes thin people, but much more often people who are fat, ask for treatment for their abnormality. Naturally the first thing to bear in mind is that both very heavy men and very light men may be perfectly healthy, so that we must not, because a man happens to be fatter than his neighbours, look upon him as diseased and therefore as requiring a course of treatment. On the other hand, we need not go to the opposite extreme of refusing to reduce a man's adiposity merely because he may seem in other respects healthy, for obesity is in many respects a great drawback to a man's enjoyment of life, and in some cases to his life's utility.

In a clinical lecture recently delivered at Guy's Hospital, Dr. Hale White[1] described the manner in which reduction of weight by diet may be accomplished, and some of the precautions which must be taken in the management of such cases. There is no hocus pocus, he says, about the diet. You must give very little carbohydrate and hydrocarbon and plenty of proteid. The principle to guide you is the physiological fact that, although fat may be derived from proteid, yet proteid leads to the destruction of the fat already in the body. From this it follows that if you give sufficient proteid, and considerably diminish the amount of carbohydrates and hydrocarbons in an ordinary diet, you can cause such a destruction of body fat that the weight of the body will decrease. It is on this principle that all the reducing diets are founded. Towers-Smith was the most thorough in his regimen, for when his patients were on the most strict diet they were taking for breakfast one pound of rump steak without fat; lunch, another pound of rump steak; dinner, one pound of grilled cod and one pound of rump steak. They drank at intervals during the twenty-four hours a gallon of warm water.

The following is a diet given to a patient in the hospital under Dr. Hale White: Breakfast—1 lb. lean beef steak, 1 pint of weak tea without milk. Dinner—½ lb. of cod, ½ lb. of lean beef steak, 1 pint of water, and a little green vegetable. Supper—1 lb. lean beef steak, 1 pint of water. He did not find this diet very pleasant, but he managed it without any difficulty. He had no medicine except a mild aperient occasionally. His total loss of weight was 9½ lbs. in three weeks.

A very stout lady for seven weeks took every day two pounds of lean steak with a little toast, and no other food. She drank fairly of tea and water. In the seven weeks she lost 16 pounds.

If a proteid diet like this is to be given, the patient must be watched, and it may be taken that if he loses at a greater rate than three pounds a week too rapid a metabolism is being induced. There is no reason to hurry, and clearly it is an advantage for the body to gradually accommodate itself to its altered conditions in regard to diet. As the weight decreases a little toast or other carbohydrate may be allowed to break the monotony of the diet, and when the weight has been sufficiently reduced the patient may be kept at that weight if he always eats sparingly of bread, takes very little butter, not much milk in his tea, and avoids sweets and large quantities of beer. He should, of course, take a fair amount of exercise. The great mistake in Banting's diet was that he allowed too little fluid. It is clear that as such large quantities of the products of destructive metabolism, and all the urea that results from the proteid diet have to be got rid of, it is well to wash the tissues out thoroughly. Therefore these patients must be allowed plenty to drink—a cup of weak tea when they wake, weak tea for breakfast, a glass of water in the middle of the morning and on going to bed at night, a cup of tea in the afternoon, and a little whisky and water at lunch and dinner would hardly be too much; but many patients prefer to take less. There is no harm in a cup of black coffee after lunch and dinner, and smoking need not be given up. It is necessary to be very cautious in dieting stout patients suffering from bronchitis. Finally, a word about thyroid gland preparations. They undoubtedly lead to a loss of weight, but they cannot be taken in large quantities for long without running great risk of thyroid poisoning. As for quack remedies sold to make fat people thin they should not be touched. Their *modus operandi* too often is the production of dyspepsia, and a loss of flesh so obtained is dearly bought.

[1] Guy's Hospital Gazette.

